# Association Between CBC‐Derived Inflammatory Indicators and 28‐Day Mortality in Patients With Coronary Heart Disease and Diabetes Mellitus: A Cohort Study From the MIMIC‐IV Database

**DOI:** 10.1155/mi/9904721

**Published:** 2026-02-17

**Authors:** Guang Tu, Zhonglan Cai, Shengnan Xue, Guofeng Zhu, Min Huang

**Affiliations:** ^1^ Department of Cardiology, Lichuan People’s Hospital, No. 2 Rifeng Road, Rifeng Town, Lichuan County, Fuzhou, 344600, Jiangxi, China; ^2^ Department of Critical Care Medicine, Zhangjiajie People’s Hospital, No. 192 Guyong Road, Yongding District, Zhangjiajie, 427000, Hunan, China; ^3^ Department of Respiratory and Critical Care Medicine, The Second Affiliated Hospital of Nanchang University, No. 1 Minde Road, Nanchang, 330006, Jiangxi, China, jxndefy.cn; ^4^ Department of Cardiovascular Medicine, The Second Affiliated Hospital of Nanchang University, No. 1 Minde Road, Nanchang, 330006, Jiangxi, China, jxndefy.cn

**Keywords:** coronary heart disease, diabetes mellitus, inflammatory indices, MIMIC-IV, prognosis

## Abstract

**Background:**

Coronary heart disease (CHD) remains the leading global cause of death; concomitant diabetes mellitus (DM) doubles short‐term mortality, largely through chronic low‐grade inflammation. Inexpensive, bedside complete blood count (CBC)‐derived inflammatory indices (NLR, MLR, PLR, SII, SIRI, and AISI) predict outcomes in CHD or DM alone, but their utility in comorbid patients is unclear.

**Methods:**

Retrospective cohort study of CHD‐DM patients from Medical Information Mart for Intensive Care‐IV (MIMIC‐IV; 2008–2022), split into training (2017–2022, *n* = 1607) and validation (2008–2016, *n* = 1145) sets. Six indices (NLR, MLR, PLR, SII, SIRI, and AISI) were calculated from initial ICU CBC (48‐h mean for sensitivity analysis). Primary outcome: 28‐day mortality, Cox regression, restricted cubic splines (RCSs), receiver operating characteristic (ROC) curves, calibration plots, and SHAP‐based variable importance were used.

**Results:**

Mortality was 10.6% (training) and 11.9% (validation). All indices showed independent, dose‐dependent associations with mortality (e.g., training MLR per 1‐SD: HR = 1.49, 95% CI = 1.37–1.61), discrimination was good (training AUC 0.767, C‐index 0.752; validation AUC 0.755, C‐index 0.746), and calibration was excellent. Spearman correlation showed moderate‐to‐strong interindex correlations (e.g., MLR‐SIRI: *r* = 0.84). SHAP analysis ranked PLR and MLR as top predictive indices. Sensitivity analysis confirmed robustness.

**Conclusions:**

Six CBC‐derived indices independently predict 28‐day mortality in critically ill CHD‐DM patients, with PLR and MLR showing superior predictive weight, and can be used for rapid, cost‐free bedside risk stratification.

## 1. Introduction

Coronary heart disease (CHD) is the world’s foremost killer [1], when it coexists with diabetes mellitus (DM) the risk of acute cardiovascular events and short‐term death [2]. Chronic low‐grade inflammation is the linchpin of this synergy: Hyperglyaemia activates the AGE–RAGE–NF‐κB axis, upregulates endothelial adhesion molecules, amplifies oxidative stress, and recruits monocytes/macrophages [3], while insulin resistance sustains a sympatho‐inflammatory loop that keeps leukocytes and platelets in a primed state [4]. These processes destabilize atherosclerotic plaques, injure coronary microcirculation, and provide an “inflammatory substrate” for ischemia–reperfusion injury and malignant arrhythmias [5].

Complete blood count (CBC)–derived indices integrate the dynamic interplay of neutrophils (acute oxidative burst), lymphocytes (immune modulation), monocytes (sustained cytokine release), and platelets (thrombo‐inflammation) [6–8]. Because these cells are simultaneously activated by the diabetic milieu, their ratios or multiplicative products reflect the global inflammatory load, immune imbalance, and thrombotic propensity more sensitively than any single cell lineage [9].

Despite this theoretical advantage, evidence linking these markers to mortality in the specific CHD‐plus‐DM population is sparse, and their dose–response or threshold effects remain unexplored. Using the publicly available Medical Information Mart for Intensive Care‐IV (MIMIC‐IV) critical care database, we undertook a large retrospective cohort study to determine whether six inexpensive CBC‐derived inflammatory indices independently predict 28‐day all‐cause mortality and to identify potential risk‐stratifying thresholds in critically ill adults with both CHD and DM.

## 2. Methods

### 2.1. Data Source and Ethics

This retrospective cohort study used the MIMIC‐IV database version 3.1, a deidentified, publicly available database of electronic health records collected between 2008 and 2022 [10]. The study protocol conforms to the ethical guidelines of the 1975 Declaration of Helsinki as reflected in a priori approval by the institutional review boards of Beth Israel Deaconess Medical Center and MIT. The study followed STROBE guidelines; author G. Tu completed the CITI program (certificate 65828445) to obtain access. Data were stratified into two subsets: the training set included records from 2017 to 2022, and the validation set included records from 2008 to 2016.

### 2.2. Study Population

We identified adults (≥18 years) with CHD from the MIMIC‐IV database using ICD‐10 codes I25.10, I25.110, I25.111, I25.118, and I25.119. To reduce confounding by noncardiometabolic inflammation, we further excluded any patient with active systemic inflammatory disease (e.g., sepsis, severe acute pancreatitis, SLE, RA, IBD, or malignancy on chemotherapy). The remaining cases were then split into training (2017–2022) and validation (2008–2016) sets as detailed below.

#### 2.2.1. Training Set (2017–2022)

**Figure 1:**
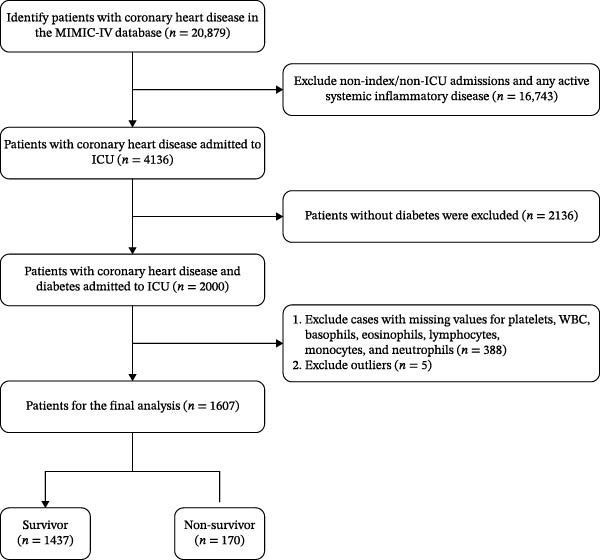
Flowchart of patient inclusion.

Initially identified 20,879 adults (≥18 years) with CHD; excluded 16,743 noninitial or non‐ICU admissions, leaving 4136 CHD patients admitted to the ICU; excluded 2136 without diabetes, resulting in 2000 patients with coexisting CHD and DM; further excluded 388 admissions with missing hematological parameters and 5 outliers (Tukey’s method), 1607 individuals remained in the training set (Figure 1).

#### 2.2.2. Validation Set (2008–2016)

Initially identified 26,617 adults (≥18 years) with CHD; excluded 21,344 noninitial or non‐ICU admissions, leaving 5273 CHD patients admitted to the ICU; excluded 3784 without diabetes, resulting in 1489 patients with coexisting CHD and DM; further excluded 339 admissions with missing hematological parameters and 5 outliers, yielding a final validation cohort of 1145 patients (1009 survivors and 136 nonsurvivors) (Supporting Information 1: Figure S1).

The specific diagnostic meanings of the ICD‐10 codes for CHD and DM are detailed in Supporting Information 2: Table S1.

### 2.3. Variables and Outcomes

Baseline demographics, BMI, and laboratory parameters were extracted for both cohorts (Table 1 for training set; Supporting Information 2: Table S2 for validation set). Six indices were calculated as follows:

**Table 1:** Baseline characteristics of study population.

Variables	Total (*n* = 1607)	Survivors (*n* = 1437)	Nonsurvivors (*n* = 170)	*p*‐value
Age, years, mean ± SD	69.2 ± 10.7	68.6 ± 10.6	73.8 ± 10.8	<0.001
Sex, *n* (%)				0.081
Female	430 (26.8)	375 (26.1)	55 (32.4)	
Male	1177 (73.2)	1062 (73.9)	115 (67.6)	
Race, *n* (%)				0.005
White	687 (42.8)	597 (41.5)	90 (52.9)	
Non‐White	920 (57.2)	840 (58.5)	80 (47.1)	
Married, *n* (%)				0.001
Married	933 (58.1)	835 (58.1)	98 (57.6)	
Divorced	157 (9.8)	145 (10.1)	12 (7.1)	
Widowed	135 (8.4)	108 (7.5)	27 (15.9)	
Single	382 (23.8)	349 (24.3)	33 (19.4)	
BMI kg/m^2^, mean ± SD	30.8 ± 9.2	30.9 ± 9.4	29.6 ± 7.7	0.096
Smoking, *n* (%)				1.000
No	1583 (98.5)	1415 (98.5)	168 (98.8)	
Yes	24 (1.5)	22 (1.5)	2 (1.2)	
WBC, × 10^9^/L, mean ± SD	10.9 ± 6.6	10.7 ± 6.2	12.6 ± 9.1	<0.001
Lymphocytes, × 10^9^/L, median (IQR)	1.6 (1.0, 2.4)	1.7 (1.1, 2.5)	1.0 (0.5, 1.5)	<0.001
Monocytes, × 10^9^/L, median (IQR)	0.5 (0.3, 0.8)	0.5 (0.3, 0.8)	0.8 (0.5, 1.1)	<0.001
Neutrophils, × 10^9^/L, mean ± SD	10.0 ± 5.7	9.8 ± 5.3	11.4 ± 7.9	<0.001
Platelets, × 10^9^/L, mean ± SD	169.6 ± 78.0	168.0 ± 73.3	183.2 ± 109.2	0.016
Lactate, mmol/L, median (IQR)	2.4 (1.8, 3.3)	2.4 (1.8, 3.2)	3.0 (2.0, 6.1)	<0.001
Sapsii, mean ± SD	38.0 ± 12.7	36.5 ± 11.4	50.8 ± 15.9	<0.001
Sofa, mean ± SD	4.9 ± 3.0	4.6 ± 2.8	6.9 ± 3.8	<0.001
Myocardial_infarct, *n* (%)				0.012
No	789 (49.1)	721 (50.2)	68 (40)	
Yes	818 (50.9)	716 (49.8)	102 (60)	
Heart_failure, *n* (%)				<0.001
No	938 (58.4)	878 (61.1)	60 (35.3)	
Yes	669 (41.6)	559 (38.9)	110 (64.7)	
Cerebrovascular_disease, *n* (%)				<0.001
No	1326 (82.5)	1208 (84.1)	118 (69.4)	
Yes	281 (17.5)	229 (15.9)	52 (30.6)	
Renal_disease, *n* (%)				< 0.001
No	1256 (78.2)	1147 (79.8)	109 (64.1)	
Yes	351 (21.8)	290 (20.2)	61 (35.9)	
NLR, median (IQR)	5.5 (3.3, 9.4)	5.2 (3.2, 8.4)	10.4 (6.0, 19.1)	<0.001
MLR, median (IQR)	0.3 (0.2, 0.6)	0.3 (0.2, 0.5)	0.8 (0.4, 1.4)	<0.001
PLR, median (IQR)	93.7 (60.2, 166.0)	87.6 (58.6, 148.6)	173.5 (98.5, 324.1)	<0.001
SII, median (IQR)	819.7 (477.5, 1576.6)	781.5 (466.2, 1406.9)	1555.6 (815.9, 3483.9)	<0.001
SIRI, median (IQR)	2.9 (1.2, 6.1)	2.6 (1.1, 5.5)	7.5 (2.8, 15.7)	<0.001
AISI, median (IQR)	437.9 (165.3, 1110.4)	393.7 (157.4, 951.4)	1115.8 (380.9, 3072.3)	<0.001

• NLR = neutrophil/lymphocyte ratio
• MLR = monocyte/lymphocyte ratio
• PLR = platelet/lymphocyte ratio
• SII = platelet × neutrophil/lymphocyte ratio
• SIRI = neutrophil × monocyte/lymphocyte ratio
• AISI = (platelet × neutrophil × monocyte)/lymphocyte ratio

For the training set, indices were derived from the first CBC obtained within 1 h of ICU entry for primary analysis (Tables 1–3) and recalculated using the mean of CBCs obtained within 48 h of ICU admission for sensitivity analysis (Table 4). For the validation set, indices were derived from the first ICU admission CBC (Supporting Information 2: Table S2–S4). Each index was categorized into cohort‐specific quartiles [11, 12]. The primary outcome was 28‐day all‐cause mortality, consistent across both cohorts. This endpoint was selected because the first 4 weeks after ICU admission represent the critical window during which inflammatory‐mediated plaque destabilization, hyperglycemic exacerbation, and thrombo‐inflammatory interactions are most pronounced in the CHD‐DM population, and during which clinicians are most likely to act on early risk‐stratification tools. The correlation matrix of all baseline covariates is depicted in Supporting Information 1: Figure S1 to illustrate potential collinearity.

**Table 2:** A multivariate Cox regression model evaluated the association between CBC‐derived inflammatory indicators and 28‐day mortality in patients with coronary heart disease complicated by diabetes.

Variable	*N*	Model 1		Model 2		Model 3	
		HR (95%CI)	*p*‐value	HR (95%CI)	*p*‐value	HR (95%CI)	*p*‐value
NLR	1607	1.03 (1.02–1.03)	<0.001	1.03 (1.02–1.04)	<0.001	1.03 (1.02–1.04)	<0.001
NLR							
Q1	402	1 (Ref)		1 (Ref)		1 (Ref)	
Q2	402	0.4 0 (0.20–0.81)	0.010	0.42 (0.21–0.84)	0.015	0.41 (0.20–0.83)	0.014
Q3	402	1.46 (0.89–2.39)	0.130	1.35 (0.82–2.22)	0.243	1.29 (0.78–2.14)	0.317
Q4	401	3.84 (2.50–5.89)	<0.001	2.58 (1.65–4.03)	<0.001	2.28 (1.45–3.57)	<0.001
MLR	1607	1.37 (1.29–1.46)	<0.001	1.51 (1.39–1.63)	<0.001	1.49 (1.37–1.61)	<0.001
MLR							
Q1	401	1 (Ref)		1 (Ref)		1 (Ref)	
Q2	402	0.71 (0.37–1.38)	0.310	0.98 (0.5–1.94)	0.962	0.94 (0.48–1.85)	0.859
Q3	402	1.79 (1.05–3.06)	0.033	1.96 (1.13–3.37)	0.016	1.86 (1.08–3.20)	0.026
Q4	402	5.15 (3.21–8.26)	<0.001	5.62 (3.43–9.21)	<0.001	4.77 (2.91–7.84)	<0.001
PLR	1607	1 (1–1)	<0.001	1 (1–1)	<0.001	1 (1–1)	<0.001
PLR							
Q1	402	1 (Ref)		1 (Ref)		1 (Ref)	
Q2	402	0.78 (0.42–1.45)	0.441	1.41 (0.74–2.69)	0.291	1.43 (0.75–2.71)	0.278
Q3	402	1.88 (1.13–3.13)	0.015	2.96 (1.73–5.05)	<0.001	2.63 (1.52–4.56)	0.001
Q4	401	4.16 (2.63–6.59)	<0.001	5.4 (3.31–8.82)	<0.001	4.78 (2.90–7.88)	<0.001
SII	1607	1 (1–1)	<0.001	1 (1–1)	<0.001	1 (1–1)	<0.001
SII							
Q1	402	1 (Ref)		1 (Ref)		1 (Ref)	
Q2	402	0.53 (0.28–0.99)	0.047	0.60 (0.32–1.13)	0.115	0.57 (0.30–1.07)	0.081
Q3	402	1.58 (0.98–2.54)	0.060	2.01 (1.22–3.29)	0.006	1.79 (1.09–2.94)	0.022
Q4	401	3.29 (2.14–5.04)	<0.001	3.48 (2.22–5.47)	<0.001	2.88 (1.83–4.54)	<0.001
SIRI	1607	1.01 (1.00–1.01)	<0.001	1.01 (1.01–1.01)	<0.001	1.01 (1.01–1.01)	<0.001
SIRI							
Q1	402	1 (Ref)		1 (Ref)		1 (Ref)	
Q2	401	0.91 (0.50–1.65)	0.756	1.36 (0.74–2.51)	0.324	1.24 (0.67–2.28)	0.498
Q3	402	1.55 (0.91–2.62)	0.103	1.71 (0.99–2.96)	0.054	1.57 (0.91–2.7)	0.106
Q4	402	4.38 (2.77–6.92)	<0.001	4.85 (3–7.86)	<0.001	4.09 (2.53–6.63)	<0.001
AISI	1607	1 (1–1)	0.028	1 (1–1)	<0.001	1 (1–1)	0.001
AISI							
Q1	402	1 (Ref)		1 (Ref)		1 (Ref)	
Q2	401	0.83 (0.46–1.5)	0.538	1.12 (0.61–2.06)	0.725	1.03 (0.56–1.89)	0.936
Q3	402	1.74 (1.05–2.89)	0.030	2.54 (1.5–4.31)	0.001	2.15 (1.27–3.63)	0.004
Q4	402	3.88 (2.46–6.1)	<0.001	4.93 (3.04–7.98)	<0.001	4.10 (2.53–6.63)	<0.001

*Note:* Model 1: unadjusted. Model 2: adjusted for age, sex, race, marital status, BMI, smoking, lactate, sapsii, and sofa. Model 3: adjusted for variables in Model 2 + myocardial infarction, heart failure, cerebrovascular disease, and renal disease.

**Table 3:** Cox regression model was used to analyze the threshold effect of CBC‐derived inflammatory indicators on 28‐day mortality.

Item	HR (95%CI)	*p*‐value
Estimate breakpoint	4.02 (3.61, 4.46)	
NLR < 4.02	0.970 (0.950, 0.990)	0.003
NLR ≧ 4.02	1.179 (1.121,1.240)	<0.001
Nonlinear test		<0.001
Estimate breakpoint	71.02 (66.85, 75.23)	
PLR < 71	0.98 (0.96, 0.99)	0.002
PLR ≧ 71	1.008 (1.005, 1.011)	<0.001
Nonlinear test		<0.001
Estimate breakpoint	576 (554, 597)	
SII < 576	0.996 (0.994, 0.999)	0.009
SII ≧ 576	1.0006 (1.0003, 1.0009)	<0.001
Nonlinear test		<0.001
Estimate breakpoint	285 (278, 293)	
AISI < 285	1.0008 (1.0004, 1.0012)	<0.001
AISI ≧ 285	1.135 (1.059, 1.216)	<0.001
Nonlinear test		<0.001

**Table 4:** A multivariate Cox regression model for sensitivity analysis: evaluating the association between CBC‐derived inflammatory indicators (calculated from 48‐h mean of ICU admission CBC) and 28‐day mortality in patients with coronary heart disease complicated by diabetes.

Variable	*N*	Model 1		Model 2		Model 3	
		HR (95%CI)	*p*‐value	HR (95%CI)	*p*‐value	HR (95%CI)	*p*‐value
NLR	1607	1.01 (1.01–1.01)	<0.001	1.01 (1.01–1.01)	<0.001	1.01 (1.01–1.01)	<0.001
NLR							
Q1	402	1 (Ref)		1 (Ref)		1 (Ref)	
Q2	402	0.60 (0.39–0.93)	0.021	0.57 (0.37–0.89)	0.012	0.55 (0.36–0.86)	0.008
Q3	402	1.40 (0.98–1.99)	0.064	1.17 (0.82–1.67)	0.398	1.12 (0.78–1.61)	0.537
Q4	401	3.07 (2.25–4.21)	<0.001	2.07 (1.5–2.87)	<0.001	1.89 (1.36–2.61)	<0.001
MLR	1607	1.20 (1.16–1.24)	<0.001	1.21 (1.17–1.26)	<0.001	1.20 (1.15–1.25)	<0.001
MLR							
Q1	401	1 (Ref)		1 (Ref)		1 (Ref)	
Q2	402	0.74 (0.47–1.14)	0.168	0.79 (0.51–1.22)	0.284	0.75 (0.48–1.16)	0.200
Q3	402	1.44 (0.99–2.09)	0.056	1.23 (0.84–1.79)	0.290	1.16 (0.79–1.69)	0.453
Q4	402	3.67 (2.65–5.08)	<0.001	2.96 (2.13–4.13)	<0.001	2.63 (1.88–3.67)	<0.001
PLR	1607	1 (1–1)	<0.001	1 (1–1)	<0.001	1 (1–1)	<0.001
PLR							
Q1	402	1 (Ref)		1 (Ref)		1 (Ref)	
Q2	402	1.06 (0.69–1.62)	0.803	1.40 (0.91–2.16)	0.123	1.35 (0.88–2.09)	0.170
Q3	402	2.00 (1.37–2.91)	<0.001	2.46 (1.67–3.61)	<0.001	2.21 (1.5–3.27)	<0.001
Q4	401	3.83 (2.71–5.43)	<0.001	4.38 (3.07–6.25)	<0.001	3.84 (2.67–5.52)	<0.001
SII	1607	1 (1–1)	<0.001	1 (1–1)	<0.001	1 (1–1)	<0.001
SII							
Q1	402	1 (Ref)		1 (Ref)		1 (Ref)	
Q2	402	0.78 (0.51–1.19)	0.241	0.78 (0.51–1.2)	0.255	0.76 (0.5–1.17)	0.216
Q3	402	1.58 (1.1–2.26)	0.013	1.61 (1.12–2.32)	0.010	1.49 (1.03–2.15)	0.033
Q4	401	3.24 (2.34–4.48)	<0.001	2.82 (2.02–3.94)	<0.001	2.48 (1.77–3.47)	<0.001
SIRI	1607	1 (1–1)	0.048	1 (1–1)	0.006	1 (1–1)	0.003
SIRI							
Q1	402	1 (Ref)		1 (Ref)		1 (Ref)	
Q2	401	1.00 (0.66–1.51)	0.992	1.12 (0.74–1.7)	0.598	1.05 (0.69–1.59)	0.814
Q3	402	1.54 (1.06–2.24)	0.025	1.44 (0.99–2.11)	0.059	1.40 (0.96–2.04)	0.084
Q4	402	3.61 (2.59–5.04)	<0.001	2.72 (1.94–3.82)	<0.001	2.41 (1.71–3.39)	<0.001
AISI	1607	1 (1–1)	0.084	1 (1–1)	0.005	1 (1–1)	0.004
AISI							
Q1	402	1 (Ref)		1 (Ref)		1 (Ref)	
Q2	401	0.96 (0.63–1.46)	0.839	1.06 (0.69–1.63)	0.777	1.00 (0.66–1.54)	0.987
Q3	402	1.82 (1.26–2.63)	0.001	1.89 (1.3–2.74)	0.001	1.80 (1.24–2.62)	0.002
Q4	402	3.54 (2.52–4.96)	<0.001	3.22 (2.28–4.55)	<0.001	2.80 (1.97–3.97)	<0.001

*Note:* Model 1: unadjusted. Model 2: adjusted for age, sex, race, marital status, BMI, smoking, lactate, sapsii, and sofa. Model 3: adjusted for variables in Model 2 + myocardial infarction, heart failure, cerebrovascular disease, and renal disease.

### 2.4. Missing Data and Statistical Methods

After excluding variables with ≥50% missingness, multiple imputation (five iterations and chained equations) was performed using the mice package for both cohorts, and imputed datasets were pooled for primary analyses. The detailed missing data distribution (missing frequency and percentage) for key variables in the training and validation sets is summarized in Supporting Information 2: Table S5. Analyses were performed in R 4.2.2 and the Free Statistics Analysis Platform 2.1.1 (Beijing) [13], with continuous variables presented as mean ± SD or median (IQR) and categorical variables as *n* (%) (consistent with Table 1 and Supporting Information 2: Table S2). Statistical analyses included multivariable Cox regression to estimate HRs and 95% CIs per 1‐SD increment and across quartiles (three nested models) for both cohorts, restricted cubic splines (RCSs) to explore nonlinear associations and thresholds, Kaplan–Meier curves with log‐rank tests to compare survival by quartile, receiver operating characteristic (ROC) curves to assess discriminative ability (with AUC and C‐index reported), calibration plots to evaluate agreement between predicted probabilities and observed mortality, Spearman’s rank correlation coefficient to analyze interindex relationships, and random survival forest (RSF) combined with SHAP analysis to quantify the relative predictive importance of each index. The proportional‐hazards assumption was tested using Schoenfeld residuals (Supporting Information 2: Table S6), and a sensitivity analysis was conducted in the training set by recalculating inflammatory indices using the mean of CBCs obtained within 48 h of ICU admission to validate the stability of the prognostic signal (Table 4).

## 3. Results

### 3.1. Patient Characteristics

After imputation, the training set (*n* = 1607) had a mean age of 69.2 ± 10.7 years; 73.2% were male and 28‐day mortality was 10.6%; nonsurvivors had higher comorbidity burdens (myocardial infarction, heart failure, and renal disease) and elevated inflammatory indices (all *p* < 0.001) (Table 1). The validation set (*n* = 1145) had a mean age of 71.4 ± 11.3 years (66.3% male) and 11.9% mortality, with similar baseline differences between survivors and nonsurvivors, including higher inflammatory indices in nonsurvivors (all *p* < 0.001) (Supporting Information 2: Table S2).

### 3.2. Multivariate Cox Regression Analysis

After full adjustment, all six indices remained independent predictors in both cohorts. In the training set, per 1‐SD increments showed HRs: NLR = 1.03, MLR = 1.49, PLR = 1.003, SII = 1.0007, SIRI = 1.01, and AISI = 1.0007 (all *p* < 0.01), showing a clear dose–response (e.g., MLR Q4 vs. Q1 HR 4.77, 95%CI 2.91–7.84) (Table 2). The validation set replicated these associations (e.g., MLR per 1‐SD: HR = 1.17; PLR Q4 vs. Q1: HR = 3.50) (Supporting Information 2: Table S3). Sensitivity analysis using 48‐h CBC means in the training set maintained predictive power (Table 4).

### 3.3. Kaplan–Meier Survival Analysis

Kaplan–Meier curves revealed stepwise decreases in 28‐day survival with increasing quartiles of all indices in both cohorts, with significant log‐rank test results (*p* < 0.001) (Figure 2, Supporting Information 1: Figure S2).

Figure 2Kaplan–Meier survival analysis curves for 28‐day all‐cause mortality. (A) Neutrophil‐to‐lymphocyte ratio (NLR); (B) Monocyte‐to‐lymphocyte ratio (MLR); (C) Platelet‐to‐lymphocyte ratio (PLR); (D) Systemic immune‐inflammation index (SII); (E) Systemic inflammation response index (SIRI); (F) Aggregate index of systemic inflammation (AISI). The log‐rank test was used for survival comparison, with *p* < 0.0001 for all indices.

**Figure:**
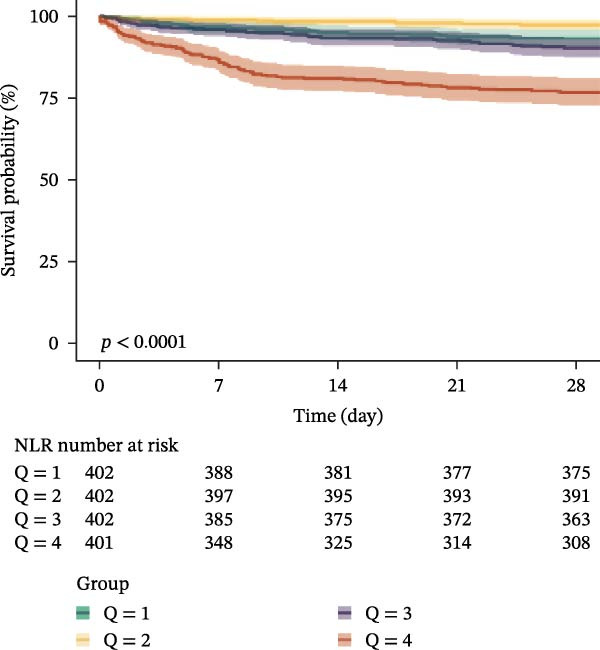
(A)

**Figure:**
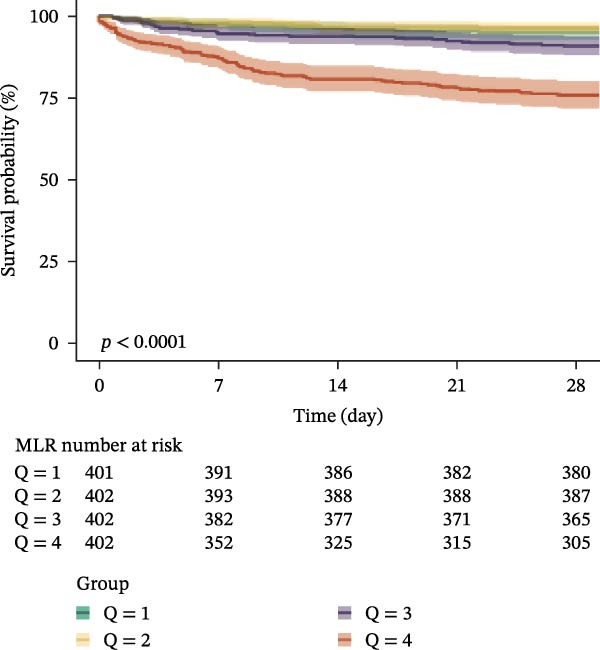
(B)

**Figure:**
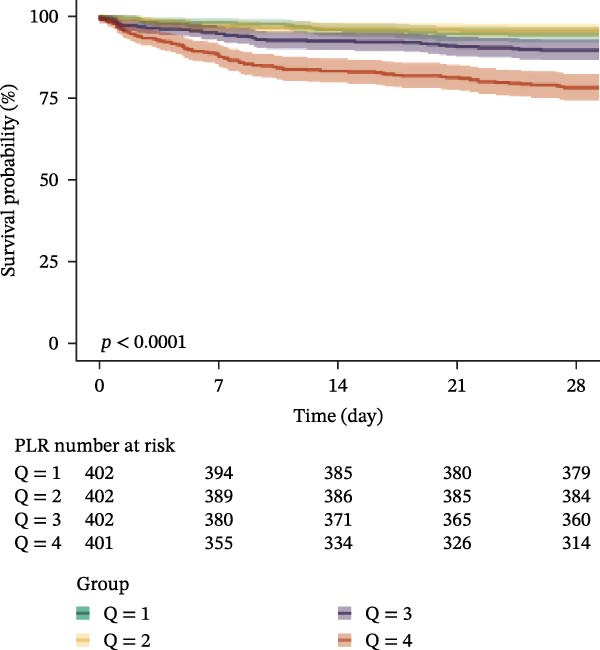
(C)

**Figure:**
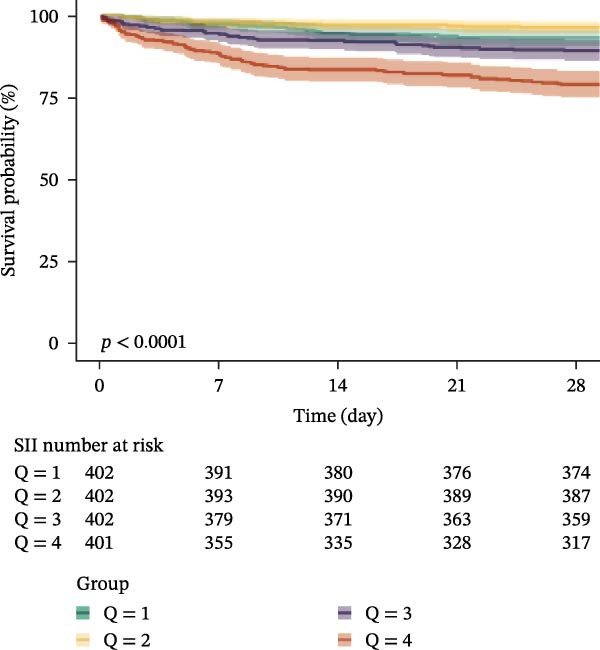
(D)

**Figure:**
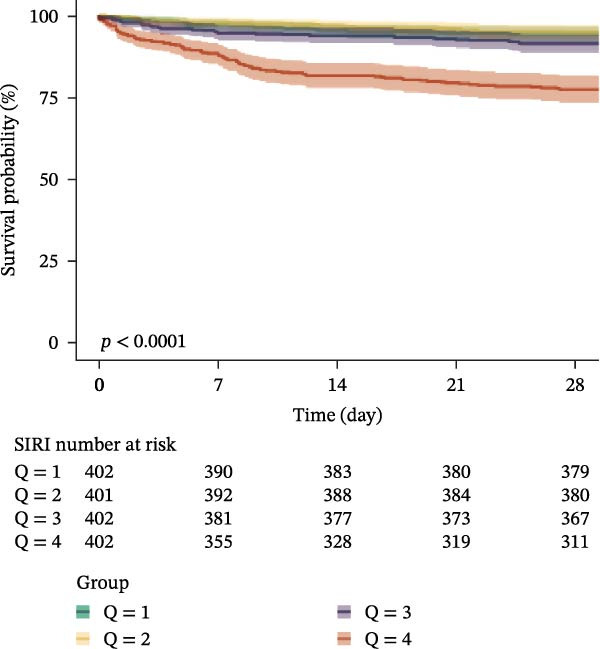
(E)

**Figure:**
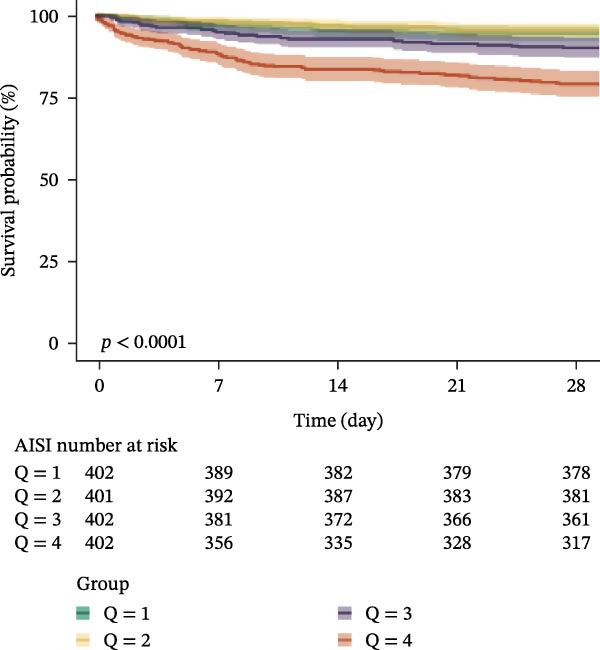
(F)

### 3.4. Nonlinear Threshold Effects

RCSs identified consistent thresholds across cohorts: NLR = 4.02, PLR = 71.02, SII = 576.00, and AISI = 285.00. Mortality rose sharply above these thresholds in the training set (all nonlinearity *p* < 0.001) (Table 3, Figure 3), and the validation set replicated this non‐linear pattern (Supporting Information 2: Table S4, Supporting Information 1: Figure S3).

Figure 3Restricted cubic spline (RCS) regression was applied to examine the association between complete blood count (CBC)‐derived indices and 28‐day mortality in patients with both coronary heart disease and diabetes. Each subplot corresponds to a specific index: (A) NLR; (B) MLR; (C) PLR; (D) SII; (E) SIRI; (F) AISI. The x‐axis represents the value of each inflammatory index, and the y‐axis represents the log hazard ratio (HR) for 28‐day mortality, with the reference value set at the median of each index.

**Figure:**
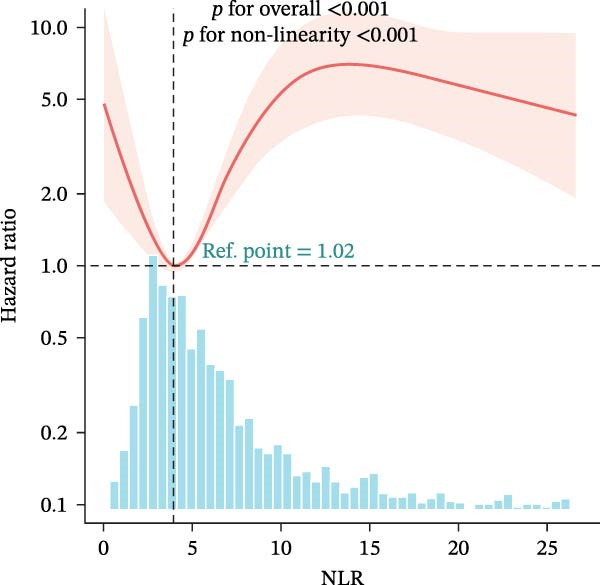
(A)

**Figure:**
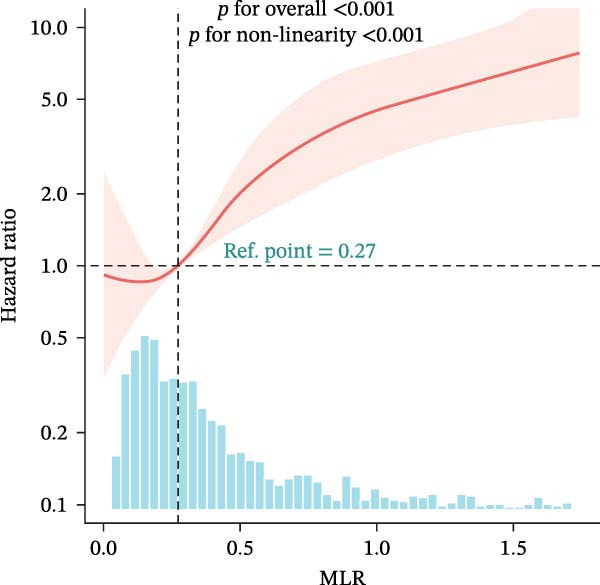
(B)

**Figure:**
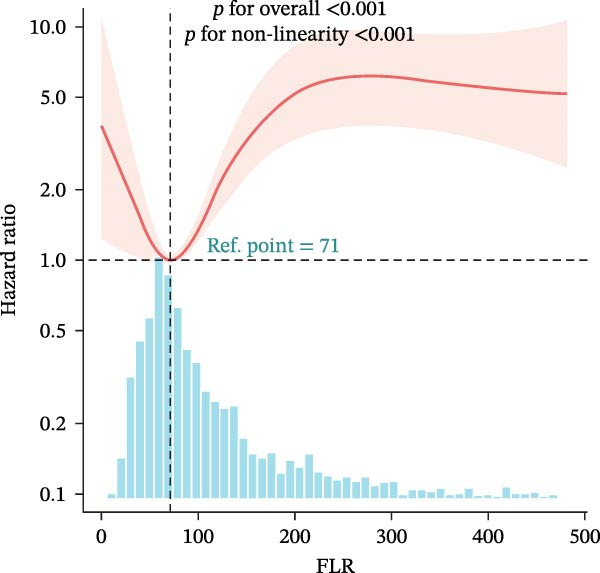
(C)

**Figure:**
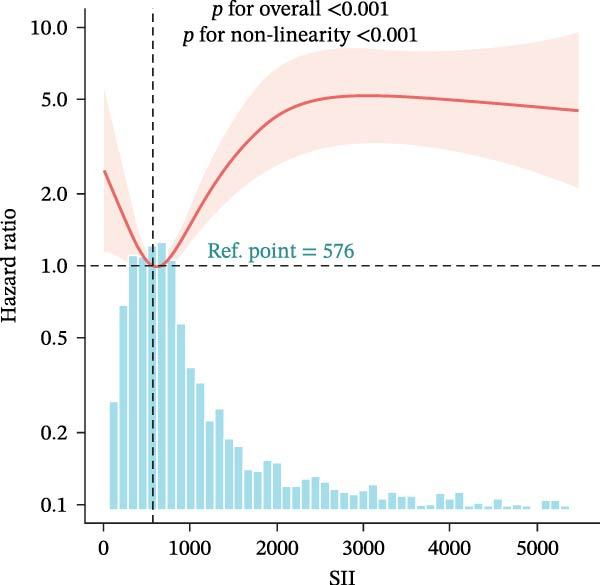
(D)

**Figure:**
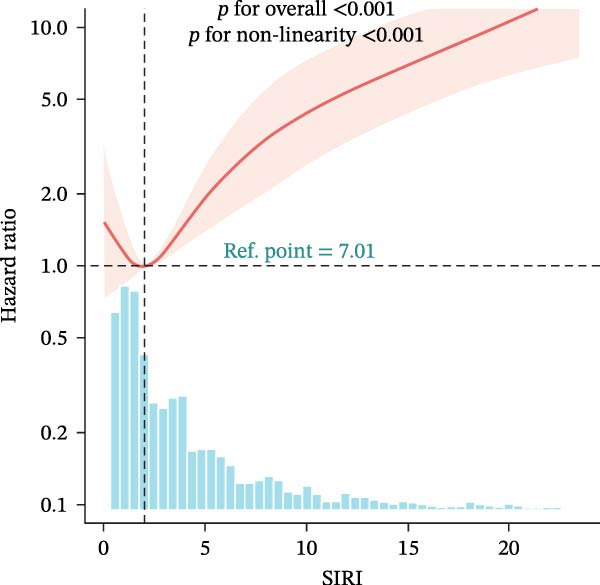
(E)

**Figure:**
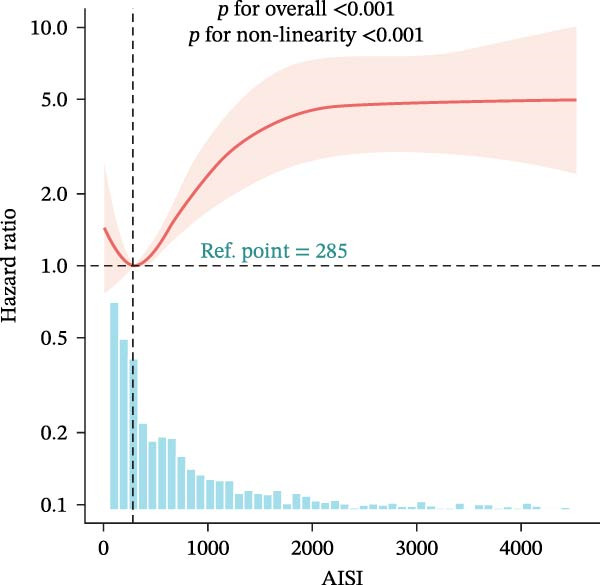
(F)

### 3.5. Sensitivity Analyses

Recalculating indices using 48‐h CBC means in the training set confirmed stable prognostic signals, with HRs consistent with primary analyses (Table 4), verifying result robustness.

### 3.6. ROC Curve Analysis

The training set AUC was 0.767 (95%CI 0.730–0.803 and C‐index 0.752), and validation set AUC = 0.755 (95%CI = 0.715–0.796 and C‐index = 0.746) indicated good discriminative ability (Figure 4).

Figure 4Receiver operating characteristic (ROC) curves of CBC‐derived inflammatory indices for 28‐day mortality. (A) Training sets. (B) Validation sets.

**Figure:**
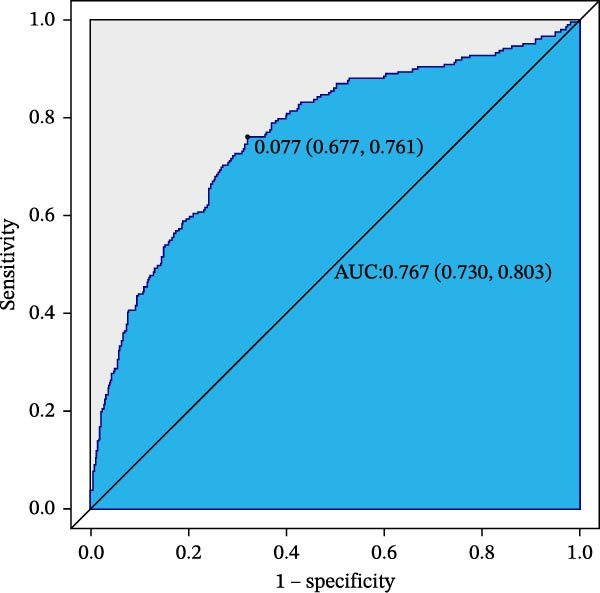
(A)

**Figure:**
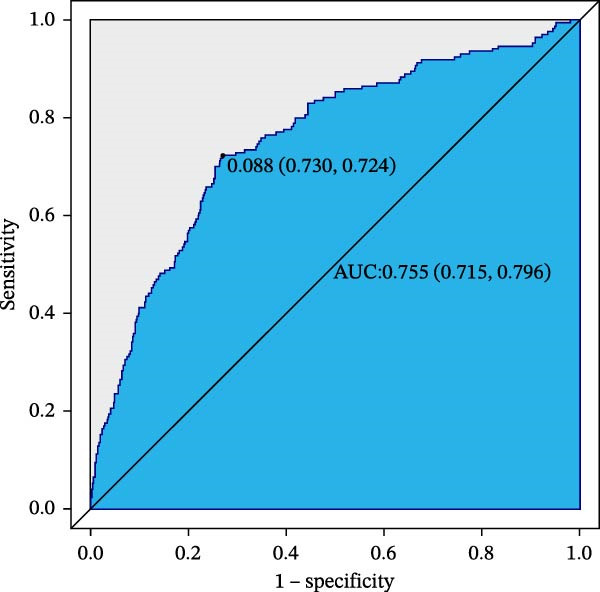
(B)

### 3.7. Calibration Curve Analysis

Calibration plots demonstrated close agreement between predicted probabilities and observed mortality in both cohorts, confirming good model calibration (Figure 5).

Figure 5Calibration plots for 28‐day mortality predicted by CBC‐derived inflammatory indices. (A) Training sets. (B) Validation sets.

**Figure:**
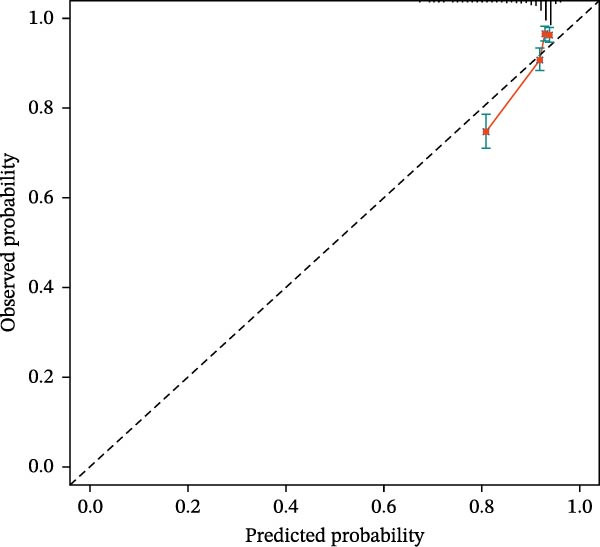
(A)

**Figure:**
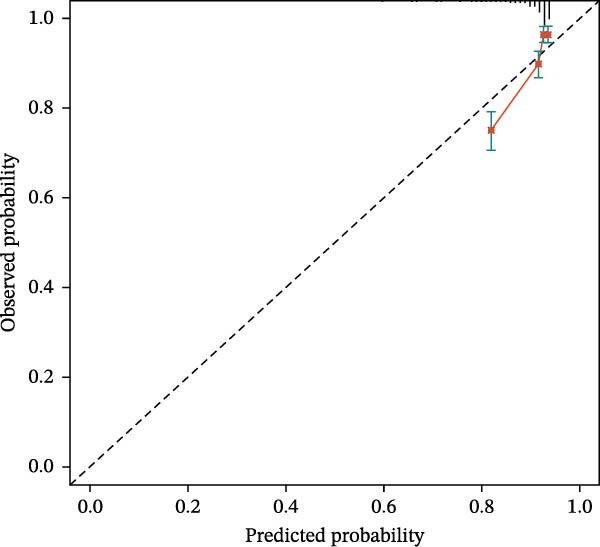
(B)

### 3.8. Correlation and Predictive Value Ranking of Inflammatory Indices

Spearman correlation analysis revealed moderate‐to‐strong interrelationships between the six indices (Figure 6A). The strongest correlations were observed between MLR and SIRI (*r* = 0.84), AISI and SII (*r* = 0.85), and SII and PLR (*r* = 0.85), while NLR showed relatively weaker correlations with AISI (*r* = 0.54). SHAP analysis (Figure 6B) quantified the relative predictive importance: PLR and MLR had the highest mean SHAP values (~0.77), followed by NLR and SII (~0.61), while SIRI and AISI showed lower but still meaningful contributions (~0.34), confirming PLR and MLR as the most informative prognostic indices.

Figure 6Prognostic value of complete blood count (CBC)‐derived indicators. Spearman correlation analysis was used to calculate correlation coefficients among CBC‐derived inflammatory indices (A). The random survival forest (RSF) method was employed to compare the predictive value of CBC‐derived inflammatory indicators for 28‐day mortality in coronary heart disease patients with diabetes (B).

**Figure:**
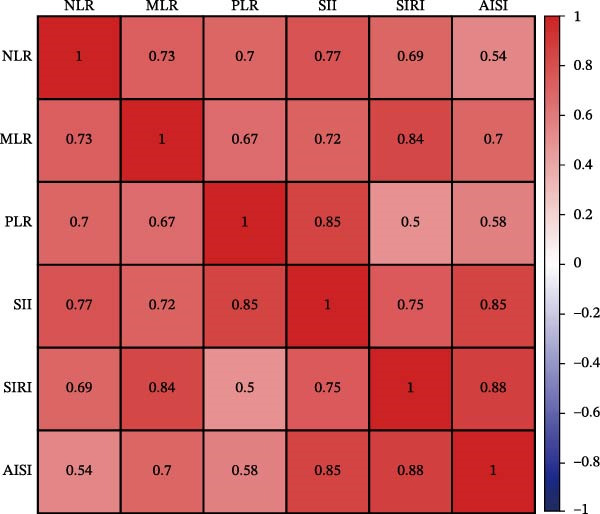
(A)

**Figure:**
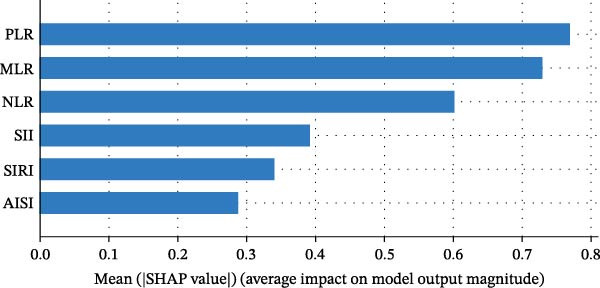
(B)

## 4. Discussion

In this large, multicenter cohort study of critically ill adults with coexisting CHD and DM, we stratified data into a training set (2017–2022, *n* = 1607) and validation set (2008–2016, *n* = 1145) to enhance generalizability. Six CBC‐derived inflammatory indices—NLR, MLR, PLR, SII, SIRI, and AISI—were independently and dose‐dependently associated with 28‐day mortality in both cohorts. Nonlinear spline analyses identified consistent, clinically actionable thresholds across training and validation sets: NLR ≈ 4.02, PLR ≈ 71.02, SII ≈ 576.00, and AISI ≈ 285.00. Importantly, the indices exhibited good discriminative ability (training set: AUC = 0.767, C‐index = 0.752; validation set: AUC = 0.755, C‐index = 0.746) and excellent calibration in both cohorts, with predicted probabilities closely matching observed mortality. The prognostic signal also persisted in a sensitivity analysis using the 48‐h CBC mean in the training set (Table 4), further confirming robustness.

Further, Spearman correlation analysis (Figure 6A) confirmed moderate‐to‐strong interindex correlations, which is biologically plausible given their shared inflammatory and thrombotic pathways. Importantly, SHAP–based predictive ranking (Figure 6B) identified PLR and MLR as the top‐performing indices, highlighting their superior ability to capture residual inflammatory risk. This aligns with our Cox regression results, where MLR showed the highest HR per SD increment, reinforcing its potential as a core bedside risk‐stratification tool.

Previous studies have linked individual CBC‐derived indices to adverse outcomes, yet three critical limitations persist. First, they typically examined CHD or DM in isolation, overlooking the amplified inflammatory interplay that occurs when both conditions coexist [14–16]. Second, most were single‐center investigations with insufficient adjustment for confounders such as renal dysfunction or prior revascularization [7, 15]. Third, they often left CBC‐derived indices ungrouped and did not explore nonlinear dose–response relationships, thereby failing to provide clinicians with actionable thresholds [6, 16]. By leveraging the 2008–2019 MIMIC‐IV multicenter ICU database, we analyzed more than 2000 well‐characterized admissions, applied rigorous multiple imputation, employed fully adjusted Cox models, and used RCSs to establish precise cut‐points—providing an externally valid and immediately applicable risk‐stratification tool.

The diabetic coronary patient is characterized by a “perfect storm” of metabolic and inflammatory insults. Chronic hyperglycemia drives the formation of advanced glycation end products (AGEs) that ligate RAGE receptors on neutrophils and monocytes, triggering NF‐κB–mediated transcription of proinflammatory cytokines and reactive oxygen species [17–20]. Simultaneously, insulin resistance increases sympathetic tone and cortisol release, leading to demargination of neutrophils and apoptosis of lymphocytes—phenomena captured by elevated NLR [21, 22]. Once primed, monocytes differentiate into macrophages that secrete IL‐6 and TNF‐α, amplifying endothelial activation and plaque vulnerability changes captured by MLR [23–25]. Platelets rendered hyperreactive by glycation and oxidative stress adhere to dysfunctional endothelium and release prothrombotic microparticles, manifesting as increased PLR, SII, SIRI, and AISI [26–28]. Collectively, these indices mirror the triad of innate immune activation, adaptive immune suppression, and thrombo‐inflammatory propensity that culminates in plaque rupture, microvascular compromise, and early death [29–31].

Our study’s strengths include a large, contemporary, multicenter ICU cohort (MIMIC‐IV) stratified into training and validation sets, validated ICD codes for CHD and DM, rigorous handling of missing data, and comprehensive adjustment for confounders. Key strengths are the comprehensive assessment of both discriminative ability (AUC: 0.767 [training] and 0.755 [validation]; C‐index: 0.752 [training] and 0.746 [validation]) and calibration—ensuring the model not only distinguishes high‐ from low‐risk patients but also provides accurate risk estimates—and the sensitivity analysis using 48‐h CBC means in the training set, which confirmed the stability of the prognostic signal independent of single‐time‐point measurement variability. By translating laboratory values available within the first ICU day into precise risk strata, our model enables clinicians to (1) identify patients at imminent risk of early death without additional cost or turnaround time; (2) prioritize resource‐intensive monitoring or escalate anti‐inflammatory/antithrombotic therapies early; and (3) incorporate a simple, reproducible metric into existing severity‐of‐illness scores, thereby enhancing shared decision‐making and family counseling.

This study has several limitations. First, the observational design precludes causal inference. Second, residual confounding (e.g., glycemic variability and antidiabetic regimens) and the predominantly North American ICU population may limit generalizability. Third, inflammatory indices were calculated from a single time point (or 48‐h mean for sensitivity), and dynamic trajectories were not assessed. Finally, the database did not distinguish type 1 from type 2 diabetes; future studies should stratify analyses by diabetes subtype to clarify potential differential effects on inflammatory markers and outcomes. Additionally, the public MIMIC‐IV extract does not contain procedural data on coronary revascularization (PCI and CABG) or infarct‐specific severity scores (e.g., TIMI and GRACE); therefore, we were unable to stratify outcomes by therapeutic intervention or by anatomical extent of myocardial infarction. We plan to address this gap by linking MIMIC‐IV to the forthcoming MIMIC‐PCI/CABG module in a follow‐up study.

## 5. Conclusion

Among critically ill adults with comorbid CHD and DM, six readily available CBC‐derived inflammatory indices independently predict 28‐day mortality across training (2017–2022) and validation (2008–2016) cohorts. Consistent threshold effects, strong discriminative ability, and excellent calibration—supported by 48‐h CBC mean sensitivity analysis and SHAP ranking highlighting PLR and MLR as top predictors—confirm their clinical utility. Incorporating these zero‐cost markers into routine care provides an immediate, accurate bedside tool to identify high‐risk patients and guide early targeted interventions.

## Author Contributions

Conception and design: Guang Tu, Guofeng Zhu, and Min Huang. Administrative support: Guang Tu and Min Huang. Provision of study materials or patients: Zhonglan Cai, Guofeng Zhu, and Min Huang. Collection and assembly of data: Guang Tu, Zhonglan Cai, and Shengnan Xue. Data analysis and interpretation: Zhonglan Cai, Shengnan Xue, and Guofeng Zhu. Manuscript writing: Guang Tu, Guofeng Zhu, and Min Huang.

## Funding

The authors received no specific funding for this work.

## Disclosure

Final approval of the manuscript was done by all authors.

## Ethics Statement

This study followed the 1964 Helsinki Declaration and its later amendments. The fully deidentified, publicly available MIMIC‐IV database was used under an institutional review board exemption granted by both MIT and Beth Israel Deaconess Medical Center, waiving informed consent.

## Conflicts of Interest

The authors declare no conflicts of interest.

## Supporting Information

Additional supporting information can be found online in the Supporting Information section.

## Supporting information


**Supporting Information 1** Figure S1: Flowchart of patient inclusion for the validation cohort. Figure S2: The Kaplan–Meier survival curves for all six inflammatory indices in the validation cohort. Figure S3: The restricted cubic spline plots examining the nonlinear relationship between each index and 28‐day mortality in the validation cohort.

**Supporting Information 2** Table S1: The ICD‐10 diagnostic codes used to identify coronary heart disease and diabetes mellitus subtypes. Table S2: The frequency and percentage of missing data for all variables in both the derivation and validation cohorts. Table S3: Schoenfeld‐residual tests confirming the proportional‐hazards assumption for each CBC‐derived inflammatory index. Table S4: The full baseline characteristics of the validation cohort, stratified by 28‐day survival status. Table S5: The multivariable Cox regression results for the validation cohort across three sequentially adjusted models. Table S6: The threshold effects of NLR and SII on 28‐day mortality identified by restricted cubic spline analysis.

## Data Availability

The MIMIC‐IV database is publicly available through PhysioNet (https://physionet.org/content/mimiciv/3.1/). All analysis codes will be provided upon reasonable request to the corresponding author.
