# Supplementary material for: Association Between CBC‐Derived Inflammatory Indicators and 28‐Day Mortality in Patients With Coronary Heart Disease and Diabetes Mellitus: A Cohort Study From the MIMIC‐IV Database
Source: Mediators Inflamm. 2026 Feb 17;2026:9904721. doi: 10.1155/mi/9904721 (PMC12913688; doi:10.1155/mi/9904721)
Supplement: Supplementary file 1 — Supporting Information 1 Figure S1: Flowchart of patient inclusion for the validation cohort. Figure S2: The Kaplan–Meier survival curves for all six inflammatory indices in the validation cohort. Figure S3: The restricted cubic spline plots examining the nonlinear relationship between each index and 28‐day mortality in the validation cohort. [file MI-2026-9904721-s001.docx]

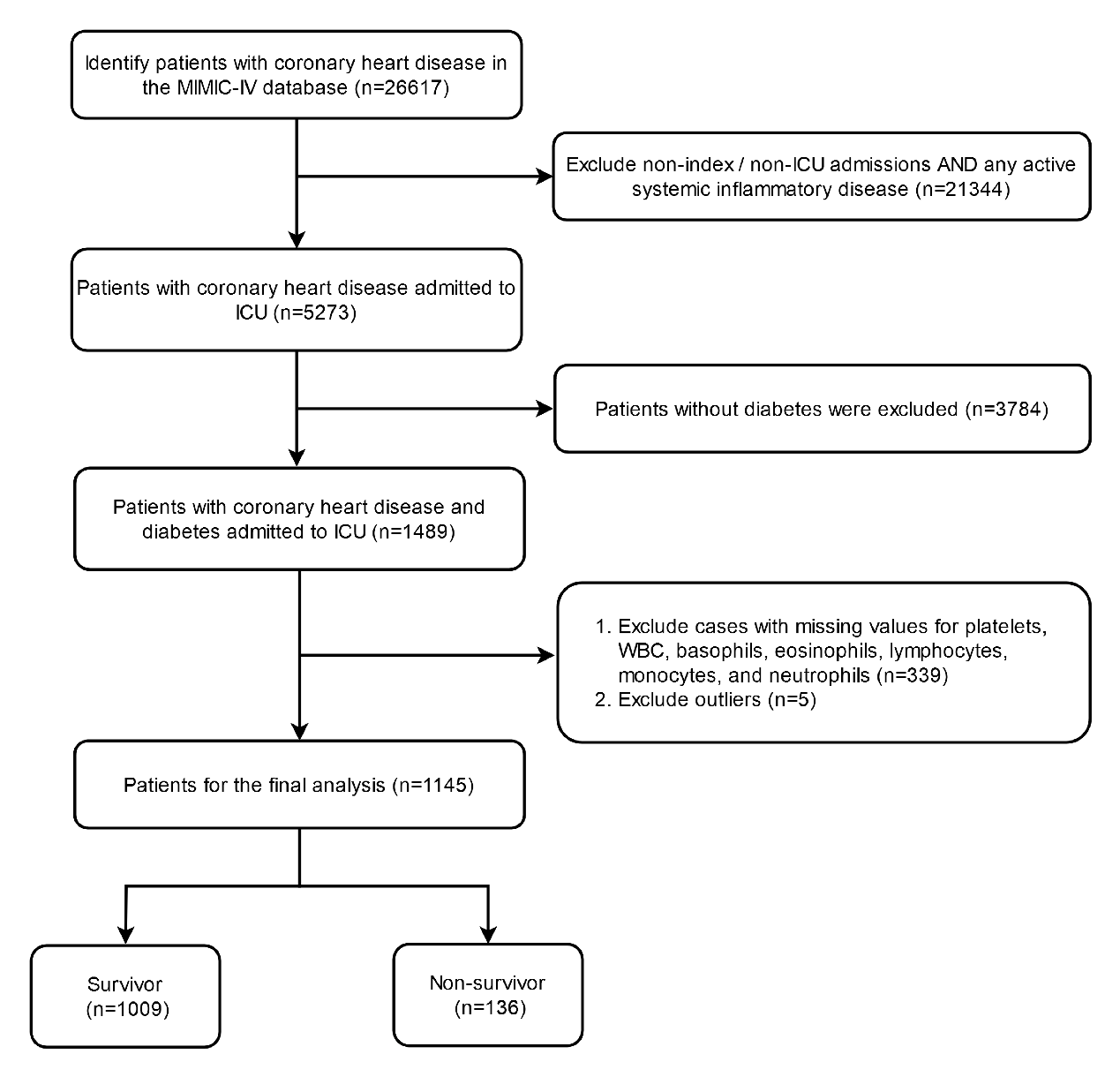


Additional Figure S1 Flowchart of patient inclusion.


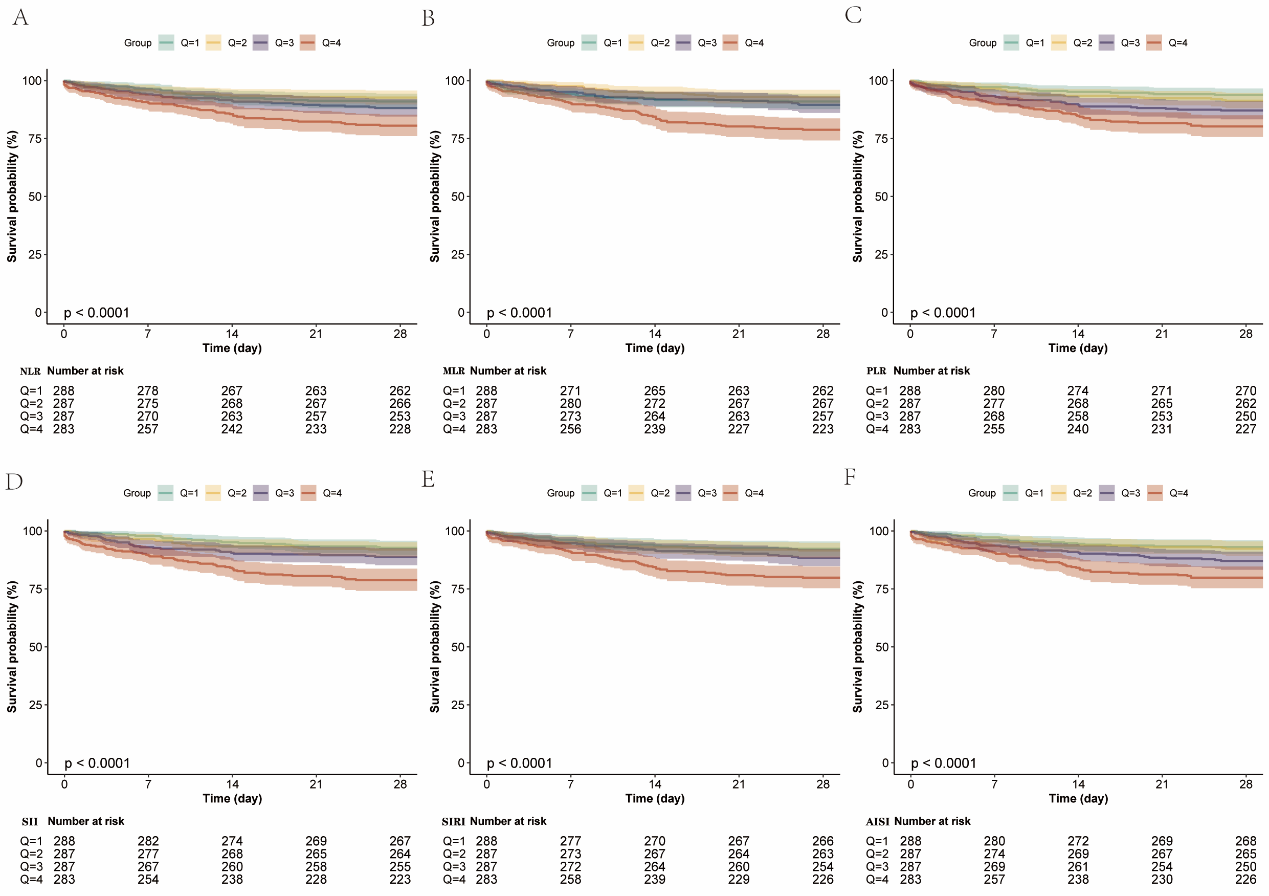


Additional Figure S2 Kaplan–Meier survival analysis curves for 28-day all-cause mortality.


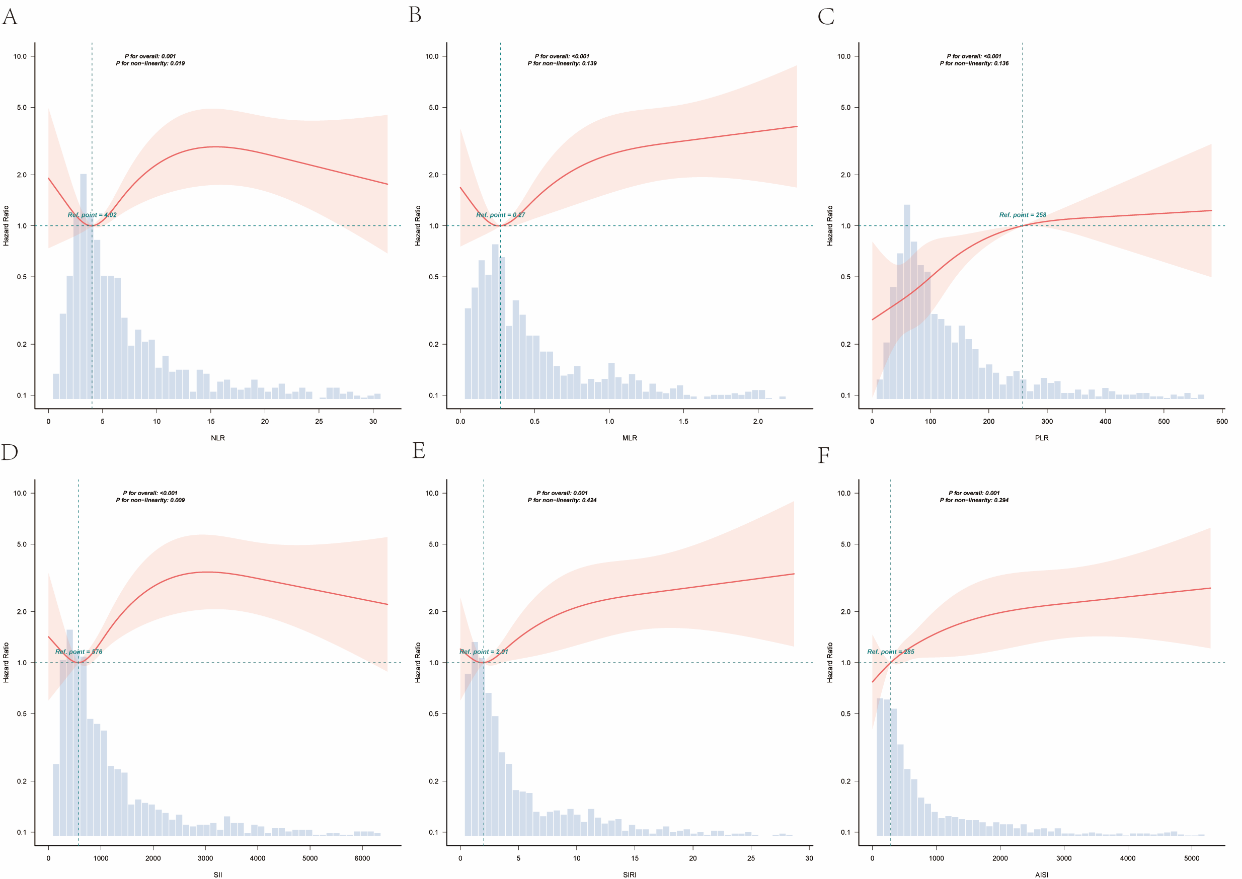


Additional Figure S3 Restricted cubic spline (RCS) regression was applied to examine the association between complete blood count (CBC)-derived indices and 28-day mortality in patients with both coronary heart disease and diabetes.
