# Supplementary material for: Association Between CBC‐Derived Inflammatory Indicators and 28‐Day Mortality in Patients With Coronary Heart Disease and Diabetes Mellitus: A Cohort Study From the MIMIC‐IV Database
Source: Mediators Inflamm. 2026 Feb 17;2026:9904721. doi: 10.1155/mi/9904721 (PMC12913688; doi:10.1155/mi/9904721)
Supplement: Supplementary file 2 — Supporting Information 2 Table S1: The ICD‐10 diagnostic codes used to identify coronary heart disease and diabetes mellitus subtypes. Table S2: The frequency and percentage of missing data for all variables in both the derivation and validation cohorts. Table S3: Schoenfeld‐residual tests confirming the proportional‐hazards assumption for each CBC‐derived inflammatory index. Table S4: The full baseline characteristics of the validation cohort, stratified by 28‐day survival status. Table S5: The multivariable Cox regression results for the validation cohort across three sequentially adjusted models. Table S6: The threshold effects of NLR and SII on 28‐day mortality identified by restricted cubic spline analysis. [file MI-2026-9904721-s002.docx]

Additional Table S1: The meaning of ICD codes for disease

| Variables | Diagnostic code (icdCode) | Meaning of diagnostic code |
| --- | --- | --- |
| Coronary Heart Disease | I2510 | Atherosclerotic heart disease of native coronary artery without angina pectoris |
|  | I25110 | Atherosclerotic heart disease of native coronary artery with unstable angina pectoris |
|  | I25111 | Atherosclerotic heart disease of native coronary artery with angina pectoris with documented spasm |
|  | I25118 | Atherosclerotic heart disease of native coronary artery with other forms of angina pectoris |
|  | I25119 | Atherosclerotic heart disease of native coronary artery with unspecified angina pectoris |
| Type 1 Diabetes Mellitus | E10.0-E10.9 | Insulin-dependent diabetes with various complications (ketoacidosis, nephropathy, retinopathy, neuropathy) |
| Type 2 Diabetes Mellitus | E11.0-E11.9 | Non-insulin-dependent diabetes with various complications |
| Malnutrition-related Diabetes | E12.0-E12.9 | Diabetes secondary to protein-energy malnutrition |
| Other Specified Diabetes | E13.0-E13.9 | Diabetes due to genetic defects, diseases of exocrine pancreas, drug-induced, and other causes |
| Unspecified Diabetes | E14.0-E14.9 | Diabetes without specified type |

Additional Table S2 Missing-data summary for the derivation (training) and validation sets

| Variable | Miss.freq(A) | Miss.percentage%(A) | Miss.freq(B) | Miss.percentage%(B) |
| --- | --- | --- | --- | --- |
| Age | 0 | 0 | 0 | 0 |
| Sex | 0 | 0 | 0 | 0 |
| Race | 0 | 0 | 0 | 0 |
| Married | 322 | 19.9752 | 39 | 3.3913 |
| Height | 454 | 28.1638 | 449 | 39.0435 |
| Weight | 15 | 0.9305 | 15 | 1.3043 |
| Smoking | 0 | 0 | 0 | 0 |
| WBC | 0 | 0 | 0 | 0 |
| Monocytes | 0 | 0 | 0 | 0 |
| Neutrophils | 0 | 0 | 0 | 0 |
| Lymphocytes | 0 | 0 | 0 | 0 |
| Platelets | 0 | 0 | 0 | 0 |
| Lactate | 463 | 28.7221 | 413 | 35.913 |
| Sapsii | 0 | 0 | 0 | 0 |
| Sofa | 0 | 0 | 2 | 0.1739 |
| Myocardial_infarct | 0 | 0 | 0 | 0 |
| Heart_failure | 0 | 0 | 0 | 0 |
| Cerebrovascular_disease | 0 | 0 | 0 | 0 |
| Renal_disease | 0 | 0 | 0 | 0 |

Note. A: training sets; B: validation sets

Additional Table S3 Proportional-hazards assumption test (Schoenfeld residuals) for CBC-derived inflammatory indicators

| Variable | Chisq(A) | *P*_value(A) | Chisq(B) | *P*_value(B) |
| --- | --- | --- | --- | --- |
| NLR | 0.017 | 0.898 | 0.859 | 0.354 |
| MLR | 0.054 | 0.816 | 0.112 | 0.738 |
| PLR | 0.884 | 0.347 | 1.601 | 0.206 |
| SII | 0.538 | 0.463 | 0.025 | 0.873 |
| SIRI | 0.518 | 0.472 | 0.514 | 0.474 |
| AISI | 0.001 | 0.974 | 0.012 | 0.913 |
| GLOBAL | 5.777 | 0.449 | 10.09 | 0.121 |

Note. A: training sets; B: validation sets

Additional Table S4 Baseline characteristics of study population

| Variables | Total  (n = 1145) | Survivors  (n = 1009) | Non-survivors  (n = 136) | *P* _value |
| --- | --- | --- | --- | --- |
| Age, years, Mean ± SD | 71.4 ± 11.3 | 70.6 ± 11.1 | 77.6 ± 10.8 | < 0.001 |
| Sex, n (%) |  |  |  | 0.542 |
| Female | 386 (33.7) | 337 (33.4) | 49 (36) |  |
| Male | 759 (66.3) | 672 (66.6) | 87 (64) |  |
| Race, n (%) |  |  |  | 0.574 |
| White | 378 (33.0) | 336 (33.3) | 42 (30.9) |  |
| Non-White | 767 (67.0) | 673 (66.7) | 94 (69.1) |  |
| Married, n (%) |  |  |  | < 0.001 |
| Married | 616 (53.8) | 551 (54.6) | 65 (47.8) |  |
| Divorced | 94 ( 8.2) | 87 (8.6) | 7 (5.1) |  |
| Widowed | 169 (14.8) | 133 (13.2) | 36 (26.5) |  |
| Single | 266 (23.2) | 238 (23.6) | 28 (20.6) |  |
| BMI, kg/m^2^, Mean ± SD | 30.4 ± 7.0 | 30.4 ± 6.8 | 30.3 ± 8.0 | 0.824 |
| smoking, n (%) |  |  |  | 0.774 |
| No | 1115 (97.4) | 983 (97.4) | 132 (97.1) |  |
| Yes | 30 ( 2.6) | 26 (2.6) | 4 (2.9) |  |
| WBC, ×10⁹/L, Mean ± SD | 10.5 ± 5.5 | 10.3 ± 5.4 | 12.0 ± 5.8 | < 0.001 |
| Lymphocytes, ×10⁹/L, Median (IQR) | 1.5 (0.9, 2.4) | 1.6 (1.0, 2.4) | 1.1 (0.6, 2.1) | < 0.001 |
| Monocytes, ×10⁹/L, Median (IQR) | 0.6 (0.3, 0.9) | 0.6 (0.3, 0.9) | 0.7 (0.4, 1.0) | 0.011 |
| Neutrophils, ×10⁹/L, Median (IQR) | 8.7 (6.1, 11.9) | 8.6 (6.1, 11.6) | 9.5 (6.7, 12.5) | 0.036 |
| Platelets, ×10⁹/L, Mean ± SD | 173.8 ± 83.2 | 171.1 ± 81.5 | 193.8 ± 92.9 | 0.003 |
| Lactate, mmol/L, Median (IQR) | 2.4 (1.7, 3.2) | 2.4 (1.7, 3.1) | 3.0 (1.9, 4.6) | < 0.001 |
| Sapsii, Mean ± SD | 38.2 ± 12.1 | 36.9 ± 11.3 | 47.9 ± 13.7 | < 0.001 |
| Sofa, Mean ± SD | 4.8 ± 2.9 | 4.6 ± 2.8 | 6.2 ± 3.5 | < 0.001 |
| Myocardial_infarct, n (%) |  |  |  | 0.037 |
| No | 576 (50.3) | 519 (51.4) | 57 (41.9) |  |
| Yes | 569 (49.7) | 490 (48.6) | 79 (58.1) |  |
| Heart_failure, n (%) |  |  |  | < 0.001 |
| No | 635 (55.5) | 592 (58.7) | 43 (31.6) |  |
| Yes | 510 (44.5) | 417 (41.3) | 93 (68.4) |  |
| Cerebrovascular_disease, n (%) |  |  |  | 0.073 |
| No | 954 (83.3) | 848 (84) | 106 (77.9) |  |
| Yes | 191 (16.7) | 161 (16) | 30 (22.1) |  |
| Renal_disease, n (%) |  |  |  | 0.001 |
| No | 792 (69.2) | 714 (70.8) | 78 (57.4) |  |
| Yes | 353 (30.8) | 295 (29.2) | 58 (42.6) |  |
| NLR, Median (IQR) | 5.5 (3.2, 10.1) | 5.2 (3.2, 9.4) | 8.3 (4.1, 16.8) | < 0.001 |
| MLR, Median (IQR) | 0.4 (0.2, 0.8) | 0.3 (0.2, 0.7) | 0.6 (0.3, 1.4) | < 0.001 |
| PLR, Median (IQR) | 101.5 (61.2, 185.2) | 95.8 (59.6, 173.1) | 162.5 (92.2, 316.5) | < 0.001 |
| SII, Median (IQR) | 857.8 (464.7, 1767.6) | 813.7 (448.1, 1560.0) | 1442.5 (613.6, 3177.2) | < 0.001 |
| SIRI, Median (IQR) | 2.9 (1.4, 7.6) | 2.7 (1.3, 6.5) | 5.1 (2.1, 15.0) | < 0.001 |
| AISI, Median (IQR) | 464.4 (198.8, 1299.7) | 430.3 (185.3, 1164.8) | 833.3 (385.9, 2729.6) | < 0.001 |

Additional Table S5 A multivariate Cox regression model evaluated the association between CBC-Derived inflammatory indicators and 28-day mortality in patients with coronary heart disease complicated by diabetes

| Variable | N | Model 1 | | Model 2 | | Model 3 | |
| --- | --- | --- | --- | --- | --- | --- | --- |
|  |  | HR (95%CI) | *P* _value | HR (95%CI) | *P* _value | HR (95%CI) | *P* _value |
| NLR | 1145 | 1.01 (1~1.01) | <0.001 | 1.01 (1~1.01) | <0.001 | 1.01 (1~1.01) | 0.001 |
| NLR |  |  |  |  |  |  |  |
| Q1 | 288 | 1(Ref) |  | 1(Ref) |  | 1(Ref) |  |
| Q2 | 287 | 0.81 (0.46~1.44) | 0.472 | 0.71 (0.4~1.28) | 0.254 | 0.69 (0.39~1.25) | 0.224 |
| Q3 | 287 | 1.33 (0.8~2.22) | 0.270 | 1.08 (0.64~1.81) | 0.781 | 1.06 (0.62~1.78) | 0.839 |
| Q4 | 283 | 2.28 (1.43~3.64) | 0.001 | 1.7 (1.06~2.73) | 0.029 | 1.63 (1.01~2.62) | 0.044 |
|  | | |  |  |  |  |  |
| MLR | 1145 | 1.16 (1.1~1.22) | <0.001 | 1.19 (1.12~1.27) | <0.001 | 1.17 (1.1~1.25) | <0.001 |
| MLR |  |  |  |  |  |  |  |
| Q1 | 288 | 1(Ref) |  | 1(Ref) |  | 1(Ref) |  |
| Q2 | 287 | 0.75 (0.42~1.35) | 0.340 | 0.7 (0.39~1.26) | 0.233 | 0.64 (0.36~1.16) | 0.139 |
| Q3 | 287 | 1.15 (0.68~1.95) | 0.595 | 0.76 (0.44~1.3) | 0.312 | 0.71 (0.41~1.23) | 0.219 |
| Q4 | 283 | 2.47 (1.56~3.92) | <0.001 | 1.74 (1.09~2.79) | 0.021 | 1.57 (0.98~2.52) | 0.058 |
|  | | |  |  |  |  |  |
| PLR | 1145 | 1 (1~1) | <0.001 | 1 (1~1) | <0.001 | 1 (1~1) | <0.001 |
| PLR |  |  |  |  |  |  |  |
| Q1 | 288 | 1(Ref) |  | 1(Ref) |  | 1(Ref) |  |
| Q2 | 287 | 1.41 (0.77~2.58) | 0.269 | 1.52 (0.82~2.79) | 0.182 | 1.44 (0.78~2.68) | 0.247 |
| Q3 | 287 | 2.15 (1.22~3.77) | 0.008 | 2.26 (1.27~4.01) | 0.006 | 2.06 (1.15~3.7) | 0.015 |
| Q4 | 283 | 3.41 (2~5.8) | <0.001 | 3.99 (2.31~6.9) | <0.001 | 3.5 (1.99~6.14) | <0.001 |
|  | | |  |  |  |  |  |
| SII | 1145 | 1 (1~1) | <0.001 | 1 (1~1) | <0.001 | 1 (1~1) | <0.001 |
| SII |  |  |  |  |  |  |  |
| Q1 | 288 | 1(Ref) |  | 1(Ref) |  | 1(Ref) |  |
| Q2 | 287 | 1.12 (0.62~2.02) | 0.718 | 1.01 (0.56~1.84) | 0.966 | 1.03 (0.57~1.87) | 0.925 |
| Q3 | 287 | 1.58 (0.91~2.74) | 0.103 | 1.33 (0.76~2.33) | 0.321 | 1.3 (0.74~2.28) | 0.363 |
| Q4 | 283 | 3.18 (1.93~5.22) | <0.001 | 2.63 (1.57~4.39) | <0.001 | 2.43 (1.45~4.07) | 0.001 |
|  |  |  |  |  |  |  |  |
| SIRI | 1145 | 1 (1~1) | 0.421 | 1 (1~1) | 0.196 | 1 (1~1) | 0.143 |
| SIRI |  |  |  |  |  |  |  |
| Q1 | 288 | 1(Ref) |  | 1(Ref) |  | 1(Ref) |  |
| Q2 | 287 | 1.1 (0.62~1.96) | 0.744 | 1.06 (0.59~1.89) | 0.844 | 1.03 (0.58~1.84) | 0.919 |
| Q3 | 287 | 1.53 (0.89~2.62) | 0.124 | 1.31 (0.76~2.26) | 0.324 | 1.34 (0.77~2.31) | 0.298 |
| Q4 | 283 | 2.81 (1.72~4.59) | <0.001 | 1.79 (1.07~2.98) | 0.026 | 1.68 (1.01~2.8) | 0.047 |
|  |  |  |  |  |  |  |  |
| AISI | 1145 | 1 (1~1) | 0.375 | 1 (1~1) | 0.121 | 1 (1~1) | 0.083 |
| AISI |  |  |  |  |  |  |  |
| Q1 | 288 | 1(Ref) |  | 1(Ref) |  | 1(Ref) |  |
| Q2 | 287 | 1.11 (0.61~2.04) | 0.725 | 1.1 (0.6~2.02) | 0.756 | 1.09 (0.59~2) | 0.789 |
| Q3 | 287 | 1.91 (1.11~3.29) | 0.019 | 1.61 (0.93~2.78) | 0.091 | 1.66 (0.96~2.89) | 0.072 |
| Q4 | 283 | 3.13 (1.88~5.21) | <0.001 | 2.42 (1.43~4.11) | 0.001 | 2.18 (1.28~3.7) | 0.004 |

Model 1: unadjusted

Model 2: adjusted for age, sex, race, marital status, BMI, smoking, lactate, sapsii, and sofa

Model 3: adjusted for variables in Model 2 + myocardial infarction, heart failure, cerebrovascular disease, and renal disease

Additional Table S6 Cox regression model was used to analyze the threshold effect of CBC-Derived inflammatory indicators on 28-day mortality

| Item | HR (95%CI) | *P* _value |
| --- | --- | --- |
| Estimate Breakpoint | 4.02 (3.80, 4.24) |  |
| NLR <4.02 | 0.970 (0.950,0.990) | 0.003 |
| NLR ≧4.02 | 1.101 (1.041,1.165) | < 0.001 |
| Non-linear Test |  | < 0.001 |
| Estimate Breakpoint | 576 (555, 596) |  |
| SII <576 | 0.996(0.994,0.999) | 0.009 |
| SII ≧576 | 1.0006 (1.0003,1.0009) | < 0.001 |
| Non-linear Test |  | < 0.001 |
